# Cardio-Pulmonary Features of Long COVID: From Molecular and Histopathological Characteristics to Clinical Implications

**DOI:** 10.3390/ijms26167668

**Published:** 2025-08-08

**Authors:** Giovanni Cimmino, Saverio D’Elia, Mariarosaria Morello, Gisella Titolo, Ettore Luisi, Achille Solimene, Chiara Serpico, Stefano Conte, Francesco Natale, Francesco S. Loffredo, Andrea Bianco, Paolo Golino

**Affiliations:** 1 Cardiology Unit, AOU Luigi Vanvitelli, 80138 Naples, Italy; 2 Department of Translational Medical Sciences, Section of Cardiology, University of Campania Luigi Vanvitelli, 80131 Naples, Italy; 3 Department of Advanced Medical and Surgical Sciences, University of Campania “Luigi Vanvitelli”, Piazza Luigi Miraglia, 2, 80138 Naples, Italy; 4 Vanvitelli Lung Unit, Monaldi Hospital, 80131 Naples, Italy; 5 Vanvitelli Cardiology and Intensive Care Unit, Monaldi Hospital, 80131 Naples, Italy; 6 Department of Life Science, Health, and Health Professions, Link Campus University, 00165 Rome, Italy

**Keywords:** long COVID, cardiovascular involvement, pulmonary fibrosis, endothelial dysfunction, molecular diagnostics, histopathology, chronic inflammation, thrombosis

## Abstract

Long COVID is a persistent post-viral syndrome with the significant involvement of both the cardiovascular and pulmonary systems, often extending well beyond the acute phase of SARS-CoV-2 infection. Emerging evidence has highlighted a spectrum of chronic alterations, including endothelial dysfunction, microvascular inflammation, perivascular fibrosis, and in some cases, the persistence of viral components in the cardiac and pulmonary tissues. At the molecular level, a sustained inflammatory milieu—characterized by elevated pro-inflammatory cytokines such as interleukin 6 (IL-6)—and chronic platelet hyperreactivity contribute to a prothrombotic state. These mechanisms are implicated in microvascular damage, cardiac strain, and impaired gas exchange, correlating with clinical manifestations such as fatigue, dyspnea, chest discomfort, and reduced exercise capacity. In certain patients, especially those who were not hospitalized during the acute phase, cardiac MRI and myocardial biopsy may reveal signs of myocardial inflammation and autonomic dysregulation. These often subclinical cardiovascular alterations underscore the need for improved diagnostic strategies, integrating molecular and histopathological markers during post-COVID evaluations. Recognizing persistent inflammatory and thrombotic activity may inform risk stratification and individualized therapeutic approaches. The interdependence between pulmonary fibrosis and cardiac dysfunction highlights the importance of multidisciplinary care. In this context, molecular and tissue-based diagnostics play a pivotal role in elucidating the long-term cardio-pulmonary sequelae of long COVID and guiding targeted interventions. Early identification and structured follow-up are essential to mitigate the burden of chronic complications in affected individuals.

## 1. Introduction

The post-acute sequelae of SARS-CoV-2 infection, commonly referred to as long COVID or post-COVID condition, has introduced a new and enduring clinical challenge on a global scale. The World Health Organization defines long COVID as a condition occurring approximately three months after the initial infection, with symptoms persisting for at least two months and not attributable to alternative diagnoses [1]. Its prevalence is estimated to range between 10% and 30%, including among those individuals who experienced only a mild acute illness [2,3]. Clinically, long COVID encompasses a wide array of symptoms—such as fatigue, cognitive impairment (“brain fog”), dyspnea, chest pain, palpitations, and reduced exercise tolerance—that reflect a multisystem involvement with a particular emphasis on cardiovascular and pulmonary dysfunction [4]. The interplay between the cardiac and pulmonary systems is central to the pathophysiology of long COVID and underpins the persistence of symptoms and increased morbidity.

Cardiovascular manifestations range from subclinical myocardial inflammation and fibrosis to myocarditis-like syndromes, often identified weeks or even months after infection via cardiac magnetic resonance imaging (CMR) [5,6]. Myocarditis-like syndromes refer to clinical presentations characterized by the symptoms and imaging findings suggestive of myocarditis, including myocardial inflammation and cardiac dysfunction, but often without a definitive histological confirmation. These syndromes may manifest as chest pain, palpitations, or elevated cardiac biomarkers, reflecting a spectrum of myocardial involvement that can be subclinical or overt. In a cohort study by Puntmann et al., cardiac abnormalities were reported in 78% of patients recovering from COVID-19, with signs of ongoing inflammation in 60% [5]. Histopathological investigations have corroborated these findings, revealing endothelial damage, perivascular infiltration by lymphocytes or macrophages, and microvascular thrombosis [7,8]. Notably, RT-PCR and in situ hybridization have occasionally detected SARS-CoV-2 RNA or proteins in the myocardial tissue, suggesting potential ongoing antigenic stimulation [7,9]. From a pulmonary perspective, persistent structural and inflammatory changes—such as organizing pneumonia, interstitial fibrosis, and capillary congestion—have been frequently documented in both biopsy and autopsy series [9,10]. Remarkably, Bharat et al. described end-stage fibrotic patterns resembling idiopathic pulmonary fibrosis in the lungs explanted from transplant recipients, supporting the rationale for lung transplantation in selected long COVID cases [10]. At the molecular level, the condition is characterized by persistent low-grade inflammation, endothelial dysfunction, and dysregulation of the coagulation cascade [11]. Elevated levels of pro-inflammatory cytokines (e.g., IL-6, interleukin (IL)-1β, tumor necrosis factor α (TNF-α), interferon γ (IFN-γ), C-reactive protein (CRP), and endothelial adhesion molecules) have been consistently reported [11,12]. These changes promote a prothrombotic environment marked by increased platelet activation [13], elevated D-dimer and plasminogen activator inhibitor-1 (PAI-1) levels [14,15], and documented cases of microthrombosis. Thromboembolic events have also been observed in the weeks to months after acute infection [11,13,14]. Endothelial dysfunction serves as a key link between inflammation and thrombosis [11,16], with chemokines such as CCL2 and neutrophil extracellular traps (NETs) implicated in the vascular injury and cardiac dysfunction observed in experimental models [12,15,16].

Histologically, the myocardial specimens from autopsy and biopsy studies demonstrate signs of chronic endotheliitis, perivascular fibrosis, and in some cases, active lymphocytic or eosinophilic myocarditis, occasionally accompanied by elevated levels of anti-cardiac antibodies. Viral RNA has been detected several months after infection [8,9]. Lung tissue analyses have also revealed persistent inflammation, alveolar type II cell senescence, vascular hyperplasia, and fibrotic remodeling up to one year post-infection [9,10].

Given the complexity and persistence of the cardio-pulmonary involvement, a multidisciplinary diagnostic approach is essential. This should integrate molecular profiling (e.g., cytokine and endothelial markers), advanced imaging modalities (MRI, CT), a histopathological assessment, and a clinical evaluation, in order to refine the risk stratification and guide targeted interventions. The identification of the persistent inflammatory and prothrombotic phenotypes may provide valuable insights for prognosis and therapeutic planning.

This review aims to synthesize the current evidence on the long-term cardio-pulmonary effects of COVID-19, focusing on the following:

- Molecular mechanisms: endothelial dysfunction, immune activation, and coagulation abnormalities;
- Histopathological findings in the heart and lung tissues;
- Clinical manifestations including myocarditis and pulmonary fibrosis;
- Implications for diagnosis, follow-up, and potential therapeutic strategies, including anticoagulants, vitamin D supplementation, and immunomodulatory agents.

Understanding these multifaceted cardio-pulmonary sequelae is crucial for developing targeted therapeutic strategies and improving patient outcomes.

Although this review focuses on the cardiovascular, endothelial, and coagulation-related mechanisms, it is crucial to recognize that long COVID is a complex and multifactorial syndrome. Among the various factors that may contribute, we find virus persistence, autoimmune diseases, chronic systemic inflammation, and residual damage to various organs. These mechanisms may interact with each other or act independently, resulting in different clinical manifestations.

## 2. Molecular Pathways in Cardio-Pulmonary Long COVID: Two Sides of the Same Coin

Long COVID is increasingly recognized as a multisystem condition, with the heart and lungs among the most frequently affected organs. Beyond the clinical manifestations, a growing body of research points to a complex web of molecular mechanisms driving persistent tissue dysfunction long after the acute phase of SARS-CoV-2 infection has resolved. Notably, the cardio-pulmonary symptoms seen in long COVID often appear to share overlapping molecular signatures, suggesting a common pathophysiological substrate involving vascular injury, chronic inflammation, and immune dysregulation.

Among the various proposed mechanisms contributing to the long COVID pathophysiology, ongoing endothelial dysfunction has emerged as a key feature. It is characterized by reduced nitric oxide (NO) bioavailability, heightened oxidative stress, and persistently elevated levels of circulating von Willebrand factor and factor VIII [17]. This endothelial damage initiates a vicious cycle, whereby the chronic inflammation further impairs the endothelial function, perpetuating a self-sustaining pro-inflammatory and pro-thrombotic environment. These alterations frequently coexist with the activation of the coagulation cascade and impaired fibrinolysis, ultimately contributing to the formation of persistent microthrombi, which have been repeatedly identified in the circulation of long COVID patients [11]. However, the clinical relevance and persistence of these microthrombi require further investigation to elucidate their exact contribution to the symptomatology.

Concomitantly, several studies have reported a sustained low-grade inflammatory state—marked by elevated levels of IL-6, TNF-α, and IL-1β [18]. While the mechanisms remain under active investigation, the evidence suggests that in a subset of patients the sustained pro-inflammatory milieu fosters autoimmune phenomena, including the production of autoantibodies targeting G-protein-coupled receptors (GPCRs), endothelial antigens, and phospholipids [19]. These autoantibodies may serve as potential diagnostic biomarkers and therapeutic targets, highlighting the importance of immune modulation in managing long COVID-related vascular dysfunction.

Of particular concern is the development of fibrotic remodeling, driven by the activation of the TGF-β signaling pathway. This has been implicated in both pulmonary fibrosis and myocardial stiffening, with potential long-term consequences for organ function [20]. Notably, fibrotic remodeling has also been documented in a subset of patients, further underscoring the pivotal role of TGF-β signaling in long COVID-related tissue damage.

Mitochondrial dysfunction and metabolic reprogramming have also been proposed to impair energy production in the cardio-pulmonary tissues, potentially contributing to hallmark symptoms such as fatigue, dyspnea, and exercise intolerance [21]. Finally, epigenetic and transcriptomic dysregulation—including altered microRNA (miRNA) expression and the persistent activation of interferon-stimulated genes—may underpin the enduring gene expression changes observed in the cardiac and pulmonary cells [22]. Therapeutic strategies targeting these epigenetic modifications could represent a promising frontier in reversing the persistent inflammation and fibrosis, offering hope for improved long-term outcomes. However, it remains to be determined whether these epigenetic and transcriptomic changes are the drivers or the consequences of the persistent cardio-pulmonary dysfunction. For instance, Cheon et al. described persistent dysregulated CD8^+^ T-cell responses in the lung tissue from post-acute COVID-19 patients, which were associated with impaired respiratory function [22].

In the sections that follow, we will delve deeper into these molecular pathways, with a particular emphasis on endothelial dysfunction as a central unifying mechanism, and explore their potential roles as diagnostic biomarkers and therapeutic targets in the context of long COVID.

### 2.1. COVID-19-Related Endothelial Dysfunction: A Common Pathway in Cardiac and Pulmonary Involvement

The endothelium is essential for maintaining vascular balance by controlling vessel tone, permeability, inflammation, and clot formation. Growing evidence points to widespread and lasting endothelial dysfunction as a key factor driving multisystem complications during both acute COVID-19 and the prolonged condition known as long COVID. Pulmonary and cardiovascular issues seem to stem from a shared mechanism—damage to the endothelium—indicating a common underlying pathophysiology. SARS-CoV-2 directly infects the endothelial cells through the ACE2 receptor and TMPRSS2 protease, leading to cellular damage and localized inflammation—a process often referred to as “viral endotheliitis” [23]. Histopathological analyses confirm a viral presence within the endothelial cells, accompanied by endothelial swelling, damage, and inflammatory cell infiltration, particularly in the pulmonary and myocardial microvasculature [23,24]. At the molecular level, COVID-19-associated endothelial dysfunction is characterized by reduced nitric oxide (NO) bioavailability, increased oxidative stress largely driven by NOX2-mediated reactive oxygen species (ROS) production, and an upregulation of adhesion molecules such as vascular cell adhesion molecule 1 (VCAM-1) and intercellular adhesion molecule 1 (ICAM-1), facilitating leukocyte adhesion and transmigration [25]. These changes promote endothelial activation with a pro-thrombotic and pro-inflammatory phenotype, further amplified by the activation of signaling pathways including NF-κB, JAK/STAT, and MAPK [26]. Disruption of intercellular junctional proteins such as VE-cadherin and ZO-1 leads to increased vascular permeability and leakage.

Circulating pro-inflammatory cytokines, including IL-6 and TNF-α, worsen the endothelial injury and maintain a harmful cross-talk between inflammation and coagulation. Complement system activation, with the deposition of components like the membrane attack complex (C5b-9) on the endothelial surfaces, contributes to microvascular inflammation and thrombosis.

In some patients, functional autoantibodies targeting ACE2 or endothelial antigens have been identified, potentially contributing to the immune-mediated endothelial damage. These autoantibodies may play a significant role, not only in sustaining vascular injury but also as diagnostic biomarkers for risk stratification. Their presence points to an autoimmune component in the long COVID pathogenesis, opening the door to targeted immunomodulatory treatments such as intravenous immunoglobulins, plasmapheresis, or therapies aimed at selective B-cell depletion. While further research is needed, these approaches could offer promising strategies to reduce vascular inflammation and improve the clinical outcomes in affected patients [19,27]. In the lungs, endothelial dysfunction increases vascular permeability, promotes alveolar edema, and plays a key role in the development of acute respiratory distress syndrome (ARDS). In the heart, endothelial injury contributes to myocardial inflammation, microvascular thrombosis, and ischemic injury, manifesting clinically as myocarditis, arrhythmias, or heart failure during the subacute and chronic phases [28,29]. Recent research suggests that endothelial dysfunction can persist for weeks or even months after the acute phase of infection, especially in patients suffering from long COVID. Biomarkers such as von Willebrand factor (vWF), angiopoietin-2, and soluble thrombomodulin (sTM) often remain elevated, while capillaroscopic and microvascular imaging studies reveal ongoing structural and functional abnormalities in the microcirculation [17,30]. Furthermore, the residual viral RNA or spike protein fragments detected in the endothelial cells imply a persistent antigen presence that may sustain local inflammation. This chronic endothelial activation might also be fueled by low-grade inflammation and interactions with activated platelets and neutrophils, including the formation of neutrophil extracellular traps (NETs), which further exacerbate the vascular injury and promote thrombosis. This chronic endothelial dysfunction likely underpins the cardinal long COVID symptoms, including fatigue, dyspnea, chest pain, and exercise intolerance, and may increase the long-term risk for cardiovascular and pulmonary complications such as myocardial infarction, stroke, and venous thromboembolism.

In conclusion, endothelial dysfunction serves as a central pathophysiological link between the cardiac and pulmonary sequelae in COVID-19. A deeper understanding of these mechanisms is essential to identify biomarkers and develop targeted therapies to improve long COVID management.

### 2.2. Immuno-Inflammatory Activation

#### Viral Endotheliitis and Interaction with the Immune System (NETs, IL-6)

One of the hallmark features of long COVID appears to be the persistence of the immune and inflammatory activation, which is often triggered by a direct viral infection in the vascular endothelium. SARS-CoV-2 is able to infect the endothelial cells through the ACE2 receptor, leading to both acute and chronic endothelial dysfunction. This results in increased vascular permeability, the upregulation of adhesion molecules such as VCAM-1 and ICAM-1, and the release of pro-inflammatory cytokines including IL-6 and TNF-α [22,24]. Importantly, endothelial cells are not just passive targets of the virus. They actively contribute to innate immunity by expressing pattern recognition receptors (PRRs) like toll-like receptors (TLRs) and RIG-I, and by secreting chemokines such as the chemokine (C-X-C motif) ligand 8 (CXCL8) and the chemokine (C-C motif) ligand 2 (CCL2), which in turn attract immune cells to the site of infection. This induces what has been described as “viral endotheliitis,” a self-sustaining cycle of inflammation and coagulation driven by cytokines like IL-6, TNF-α, and IL-1β, alongside chemokines including CXCL10 and MCP [25,31]. A particularly interesting factor here is the role of the neutrophil extracellular traps, or NETs. These networks of DNA, histones, and enzymes serve to trap pathogens during the acute phase of an infection, providing a protective effect. However, when NET formation becomes excessive and persistent—as observed in long COVID patients—they can actually damage the endothelium, promote microthrombosis, and contribute to fibrosis. Circulating complexes containing DNA-myeloperoxidase and citrullinated histones have been detected not only during the acute infection but also in long COVID, suggesting an ongoing microvascular injury [15]. Moreover, this process is amplified by inflammatory cytokines and complement activation, creating a vicious cycle of inflammation and thrombosis [12]. Among these mediators, IL-6 stands out for its pivotal role. While IL-6 is critical during the acute phase, it often remains elevated for months afterward, perpetuating the endothelial activation, reactive oxygen species (ROS) production via NADPH oxidase, and intravascular coagulation [32]. Elevated IL-6 levels have been associated with poorer clinical outcomes, persistent systemic symptoms, and delayed recovery in long COVID patients [31,32]. Interestingly, in some individuals, this immune activation does not resolve quickly but persists over six to eight months. This is characterized by the presence of activated CD14^+^ monocytes, exhausted CD8^+^ T cells, and dysfunctional natural killer (NK) cells [18]. This ongoing, low-grade inflammatory state might be driven by residual viral antigens that continue to subtly stimulate the immune system [18]. At the same time, emerging evidence points to an autoimmune component. Functional autoantibodies targeting the endothelial structures, β-adrenergic and muscarinic receptors, and components of the complement system have been identified. These autoantibodies may play a role in the autonomic dysfunction, chronic fatigue, and vascular damage frequently observed in long COVID [19,33].

These changes highlight the three key elements of Virchow’s triad—endothelial injury, blood flow stasis, and hypercoagulability. This triad serves as a crucial framework for understanding the thrombotic complications that can arise in both acute and long COVID cases.

### 2.3. Coagulation Disorders: Is COVID-19 a Thrombotic Disease?

#### 2.3.1. Molecular Mechanisms in the Persistent Prothrombotic State

In long COVID, the platelets remain activated, showing increased P-selectin and a stronger tendency to aggregate. Even more concerning, they interact with neutrophils, promoting the formation of NETs—webs of DNA and enzymes. While these NETs can trap pathogens effectively, excess NETs may damage the endothelium and trigger microthrombosis.

At the same time, chronic elevation is observed in the following:

- PAI-1, which blocks fibrinolysis;
- Tissue factor (TF), reinforcing coagulation;
- Factor VIII, often elevated in inflammation [11,34].

This combination appears to produce microthrombi that are resistant to natural breakdown, particularly in the cerebral and pulmonary microcirculation—likely drivers of persistent long COVID symptoms [34].

This instigates a dangerous positive feedback loop—driven by thrombo-inflammation—that worsens the endothelial injury and encourages further microthrombus formation [34].

#### 2.3.2. Persistence of D-Dimer and Hemostatic Markers Clinical Implications: Late Thromboembolic Events & Subclinical Ischemia

One study by Fogarty et al. reported that 25–30% of long COVID patients still showed elevated D-dimer 3–6 months post-infection—quite a striking finding, since D-dimer indicates ongoing clot breakdown [28]. They also documented increased levels of von Willebrand factor (VWF), Factor VIII, and thrombin, painting a consistent picture of a sustained prothrombotic and pro-inflammatory profile [28,35].

#### 2.3.3. Clinical Implications: Late Thromboembolic Events & Subclinical Ischemia

From a clinical perspective, these biochemical abnormalities translate into real-world risk. A massive cohort study in England and Wales (48 million adults) showed that patients with COVID-19 had up to a 30 times higher risk of a pulmonary embolism in the first weeks, and that this risk remained elevated (HR > 1.5) for up to a year [36]. Moreover, many patients seem to suffer from subclinical ischemia—silent damage to the brain, heart, or lungs—which may underpin the persistent cognitive fog, dyspnea, and chronic fatigue. These observations suggest the importance of long-term hemostatic and cardiovascular monitoring for anyone recovering from COVID-19.

Although it is well known that microthrombi and endothelium damage occur in acute COVID-19, their persistence in long COVID is still a topic of research. Current evidence is mostly based on small studies, in vitro observations, or autopsy data, and further robust clinical validations are needed.

## 3. Histopathological Evidence: Myocardial and Pulmonary Findings

The cardiovascular system is frequently involved in long COVID disease.

The molecular mechanisms behind the cardiac damage in the chronic phase are still unknown. Some studies have hypothesized the existence of persistent viral reservoirs localized in the heart that are responsible for chronic inflammation. This process can be worsened by obesity due to the secretion of adipokines (such as monocyte chemoattractant protein-1) and chemokines. This causes endothelial dysfunction mediated by reduced endothelial nitric oxide synthetase activity, the production of reactive oxygen species, and mitochondrial dysfunction [37,38]. This mechanism leads to tissue damage with chronic myocardial fibrosis, decreased ventricular compliance and contractility, reduced myocardial perfusion, increased myocardial stiffness, and also to dangerous arrhythmias.

Another explication for the cardiac chronic damage is represented by molecular mimicry which determines a reaction between the cardiac antigens and antibodies [37]. This process, together with cytokine release and increased catecholamine production, is responsible for dysautonomia resulting in tachycardia and a reduction in vascular tone [38]. Furthermore, a study conducted in June 2020 which evaluated 100 patients using CMR, demonstrated persistent myocardial inflammation 71 days after the acute infection.

Various analyses of post-COVID-19 patients utilizing positron emission tomography (PET), CT, and MRI observed persistent perfusion deficits in the heart and lungs at 40–60 days post-infection [37]. Coronaviridae family infections demonstrated severe pathological cardiac changes. In acute infections, the following were observed: severe interstitial edema, vascular congestion and dilation, and infiltration of red blood cells between the degenerative myocardial fibers. A 12-month evaluation of the same MHV1-infected mice showed, in addition to these changes, the presence of inflammatory cells and apoptotic bodies, acute myocyte necrosis, hypertrophy, fibrosis, vacuolation, and myofibril disorganization with a loss of striations [39]. Another study evaluated histological modifications in human biopsies due to myocarditis in long COVID. The most important findings were cardiomyocyte necrosis or dystrophy, interstitial edema, the infiltration of lymphohistiocytes and eosinophils, endotheliitis, microvascular and right ventricle thrombosis, and perimuscular and perivascular fibrosis [40].

The pulmonary system is the main target of SARS-CoV-2, even if it can also extend to other organs and lead to tissue injury and multiorgan failure [41]. In long COVID-19, the chronic lung involvement likely represents a complex mixture of sequelae due to the direct viral damage, acute lung injury with diffuse alveolar damage (DAD), ventilator-associated lung injury, and immune-mediated processes [42].

Histopathological changes in the lungs of COVID-19 patients are determined by the involvement of hyaline membranes, endothelial/interstitial cells, alveolar cells, type I/II pneumocytes; furthermore, interstitial and/or alveolar edema, hemorrhage, microthrombi, and fibrin depositions may also be present. There is a direct correlation between the imaging and histology of the lung changes in the areas affected by COVID-19 infection, but during autopsy it has been demonstrated that microvascular damage and thrombosis are also present in the areas that were radiologically normal on pre-mortem CT [43]. Some studies describe histological findings suggestive of pneumonia and organized pneumonia, and other studies describe diffuse interstitial lung disease that can lead to pulmonary fibrosis. There are variable degrees of fibroblast proliferation and collagen deposition with dense and myxoid fibrotic reaction patterns. Proliferative cellular desquamation is typical of acute respiratory distress syndrome (ARDS) [44,45].

Mario Culebras et al. performed a study on patients with previous severe pneumonia resulting from SARS-CoV-2, which included radiological (CT) and histopathological examinations of transbronchial lung cryobiopsy samples obtained from areas with pulmonary fibrosis. Patients presented with clinical, radiological, and pulmonary functional criteria compatible with diffuse interstitial lung diseases and displaying different histological patterns. The observed data support the existence of interstitial involvement after severe SARS-CoV-2 pneumonia and the need to commence a specific antifibrotic therapy or intervention to prevent disease progression. Transbronchial cryobiopsy has proven to be a safe technique with a high diagnostic yield in the investigation of post-COVID diffuse interstitial lung diseases [46].

An important contribution in understanding the physiological and structural consequences of SARS-CoV-2 infection comes from histopathological studies, which reveal a variety of persistent or abnormal pulmonary tissue responses associated with long COVID. In particular, transbronchial lung biopsies from the lung tissue of long COVID patients have revealed diffuse alveolar damage (DAD) with vascular abnormalities (VA), VA with an inflammatory pattern, and inflammatory and fibrotic patterns [47]; these have all been implicated as possible underlying causes of the slow recovery in long COVID patients. The endothelial dysfunction, as demonstrated by the blunted myocardial oxygenation responses obtained during stress testing, further supports the presence of a vascular component in the long COVID pathology [48]. Autopsy studies consistently show diffuse alveolar damage in the lungs of patients who die from COVID-19, with features such as capillary congestion, the necrosis of pneumocytes, hyaline membrane formation, interstitial and intra-alveolar edema, and type 2 pneumocyte hyperplasia. Platelet–fibrin thrombi in the small vessels are also common, indicating coagulopathy. Inflammatory infiltrates are mainly composed of macrophages and lymphocytes, and viral particles are found in pneumocytes [49].

In addition to the direct pulmonary parenchymal injury, recent evidence has highlighted the involvement of the bronchopulmonary lymphatic system. Fedorov et al. [50] conducted a morphological and immunohistochemical analysis of the bronchopulmonary lymph nodes in deceased COVID-19 patients. Their findings revealed an early depletion of the lymphoid follicles approximately seven days after symptom onset, suggesting the suppression of the germinal center B-cell responses. As the disease progressed, a shift toward CD8^+^ T-cell predominance was observed, with a stabilization of the CD4^+^/CD8^+^ ratio occurring at around 21 days post-symptom onset. These immunoarchitectural changes reflect a profound alteration in the local immune function, potentially contributing to impaired viral clearance, persistent inflammation, and a vulnerability to secondary infections. These mechanisms may, at least in part, underlie the development of the chronic pulmonary inflammation and fibrosis observed in the context of long COVID [50].

Furthermore, in a recent study, bronchoscopies were performed on patients with PLC who had significant symptoms but with normal pulmonary function tests (PFTs) and computed tomography (CT) images. The patients had no prior history of airway disease or a significant smoking history. The researchers obtained bronchial brush samples from the sixth to eighth generation airways and subjected the cells to single cell RNA sequencing (scRNAseq). The scRNAseq data showed an increased number of neutrophils in the airway mucosa, along with an increased expression of mucin genes, and an upregulation of interleukin (IL)-33 and T-cell receptor signaling in the secretory mucosal cells [51]. Transcriptomic pathway analysis of the PLC airway mucosa revealed a pro-inflammatory pattern with enrichment in the neutrophil-associated activation signatures, including neutrophil degranulation [51]. Overall, the pulmonary involvement in long COVID is characterized by a combination of interstitial fibrosis, vascular remodeling, and ongoing low-grade inflammation, leading to lasting functional impairment and symptoms. Why patients with PLC have evidence for ongoing small airway inflammation 1–3 years post-acute infection remains a mystery. Son et al. [52] have suggested that this is due to the generation of autoimmune antibodies in some individuals following their acute bout of SARS-CoV-2 infection [51]. Table 1 provides a summary of molecular, pathophysiologica and clinical changes observed in long COVID patients.

## 4. Myocarditis in Long COVID: Pathophysiology and Clinical Manifestation

Myocarditis and cardiomyopathy represent significant cardiovascular complications in the post-acute phase of COVID-19, contributing to a substantial burden of morbidity [53]. Studies reveal an elevated risk of long-term cardiovascular outcomes, including myocarditis, following acute infection [54]. The pathophysiology is multifactorial, involving persistent low-grade inflammation, potential viral persistence, endothelial dysfunction, and autoimmune-mediated processes [55]. These mechanisms may act synergistically, amplifying myocardial injury even in the absence of active viral replication. Chronic immune dysregulation and molecular mimicry between the viral antigens and myocardial proteins may provoke an autoimmune attack on the cardiac tissue, contributing to ongoing inflammation and tissue remodeling. This can lead to a spectrum of clinical presentations, from subclinical inflammation to overt heart failure and arrhythmias, as illustrated in various case series [56].

A distinct but related immunopathological entity is multisystem inflammatory syndrome in adults (MIS-A), which typically presents within 2–6 weeks of acute SARS-CoV-2 infection and can mimic or trigger fulminant myocarditis. In MIS-A, cardiac involvement—including ventricular dysfunction and arrhythmia—is prominent and often occurs in the context of systemic hyperinflammation with elevated ferritin, CRP, and IL-6 levels [57,58]. Beyond MIS-A, there is an emerging recognition of a prolonged or delayed multisystem inflammatory response that may persist or arise months after the acute phase, contributing to the chronic symptoms and organ dysfunction in long COVID. Although this post-acute inflammatory state is not yet formally classified under a specific syndrome, it is thought to play a role in sustained myocardial inflammation and damage in some patients [2,59].

Patients affected by the long COVID syndrome may experience symptoms such as chest pain, palpitations, fatigue, and dyspnea, which are frequently misattributed to generalized post-viral fatigue unless investigated with cardiac-specific diagnostics. Diagnostic evaluation follows established pathways typically involving electrocardiograms, cardiac troponins, and natriuretic peptides [56]. Cardiac MRI is particularly valuable due to its ability to detect myocardial edema and fibrosis via late gadolinium enhancement, which are hallmarks of myocarditis. In select cases, an endomyocardial biopsy may be considered to confirm the diagnosis and rule out other etiologies, although it is invasive and is not routinely performed [60]. Management is largely supportive and aligns with the standard heart failure guidelines, often involving beta blockers, ACE inhibitors, and mineralocorticoid receptor antagonists. Non-pharmacological strategies, including exercise restriction during the inflammatory phase and a gradual reintroduction of physical activity under supervision, are also recommended to avoid arrhythmogenic complications [61]. In cases with evidence of multisystem inflammation, immunomodulatory therapies such as corticosteroids and intravenous immunoglobulins may be considered, although the data on their efficacy in chronic post-acute inflammation remain limited. The long-term prognosis remains an area of active investigation, underscoring the importance of vigilant cardiovascular follow-up in patients with post-acute COVID-19 syndrome [54]. Longitudinal cohort studies are ongoing to determine the trajectory of cardiac recovery or deterioration over months to years. There is growing recognition that even patients with initially mild symptoms may develop structural changes that are only detectable using advanced imaging, underscoring the importance of risk stratification and early intervention [62].

## 5. Pulmonary Involvement in Long COVID

The symptoms reported by long COVID patients are fatigue, cough, cognitive deterioration (anxiety and depression), joint pain, and above all dyspnea. Numerous studies have shown an improvement in dyspnea over time in the majority of patients; however, one study conducted in Wuhan highlighted the presence of dyspnea in 14% of survivors two years after contracting the COVID-19 disease [63]. Steinbeis et al. used St. George’s Respiratory Questionnaire (SGRQ) to highlight the differences in respiratory symptoms; the symptoms in those patients with a greater disease severity in the acute phase improved in the 12 months following onset, while the symptoms in patients with a less severe disease remained almost constant during the 12-month follow-up [64]. Dyspnea after COVID-19 is due to multifactorial mechanisms that include the parenchymal sequelae, alterations in cardio-respiratory function, and muscle deconditioning.

Yan D. et al., through a multicenter questionnaire-based study, found that the most common symptom was fatigue. Fatigue is common after an acute lung injury [65]. Fatigue causes substantial impairments in physical function and the health-related quality of life. Chronic fatigue is common in other respiratory diseases, indicating that COVID-19 and other infectious diseases in the recovery phase may share a common mechanism. Advanced age was found to be a risk factor for developing long COVID. Many studies have shown that older adults are a high-risk group for long COVID; reinfection with SARS-CoV-2 also contributed to the additional risks of developing long COVID [65].

Several studies have revealed that vaccinated subjects may be less likely to report symptoms of COVID-19 in the long term. Some evidence suggests that receiving a higher number of vaccine doses is linked to a lower likelihood of developing certain symptoms. However, this protective effect seems to vary depending on the symptoms and may not apply to all cases, particularly in cardiovascular complications, as shown by recent large-scale cohort studies [65]. These findings suggest a complex interaction between the immune protection provided by the vaccine and the persistent pathophysiological processes, including endothelial damage and coagulation issues. Although vaccination has been shown to be effective in preventing severe cases of COVID-19, its role in mitigating long-standing COVID is still under investigation. In particular, the difference between the vaccine protection against general symptoms and persistent cardiovascular risk highlights the need for more in-depth, mechanistic research. These studies should examine whether endothelial dysfunction, pro-thrombotic states, and chronic inflammation may continue to occur even in the absence of viral replication, or whether current immunization strategies can adequately address them.

Regarding lung function, a restrictive ventilation pattern is often observed in long COVID patients, consistent with interstitial lung disease (ILD). Total lung capacity (TLC) and the diffusing capacity for carbon monoxide (DLCO) were impaired, especially in patients with fibrotic lesions evident using CT. Pulmonary functional outcomes are related to the severity of the initial course. Patients with a very severe COVID-19 course showed significantly more frequent signs of restrictive ventilation and diffusion impairment during the 2-year follow-up. Diffusion impairment is therefore also the most common abnormality observed after COVID-19 disease. A reduction in the transfer coefficient is found less frequently. The transfer coefficient (KCO) correlates the DLCO with the ventilated alveolar volume (DLCO/VA) and, unlike DLCO, it is not affected by a reduction in the ventilated alveolar volume, as in obesity [66]. Dana Yelin et al. [67] evaluated the reduction of DLCO in association with other risk factors including female sex, smoking, and obesity. A greater reduction in DLCO was found in women; this difference between the sexes in long COVID may be explained by hormonal effects on the regulation of the inflammatory consequences of COVID. The association between cigarette smoking and impaired diffusion is a long-term consequence of the parenchymal insult delivered by smoking-related toxicants that results in a reduced diffusion capacity. The inverse association between BMI and DLCO is in line with previous findings [68] not related to COVID-19. DLCO may be increased in individuals with extremely severe obesity. It is possible that the presumed protective role of BMI is simply related to a higher baseline DLCO in these individuals. Radiological changes are often detected in long COVID patients, which usually improve over time. The lesions found are fibrotic lesions such as reticulations, traction bronchiectasis, and honeycomb lesions. CT changes showing greater lung involvement were found predominantly in those patients with moderate DLCO impairment and severe restriction. Fibrotic changes are significantly reduced in patients with mild restriction and diffusion impairment and in those without restriction and diffusion impairment. In a study conducted 12 months after hospitalization with COVID-19, fibrotic sequelae were visualized using high-resolution computed tomography (HR-CT) in only 1% of patients. In the majority of cases, non-fibrotic changes with a limited anatomical extension were observed. In the 2-year follow-up of patients with severe COVID-19 pneumonia, a marked reduction in the ground-glass opacities (GGO) was shown; reticular lesions remained the predominant clinical picture, without the presence of traction bronchiectasis and honeycomb lesions [69]. In long COVID patients, pulmonary capillary vasculopathy is present. Some studies using dual-energy CT examination have confirmed a persistent microangiopathy with perfusion defects due to the presence of fibrinoid thrombi, confirming that endothelial damage is a central process in COVID-19 pathogenesis, both in the lungs and in other parts of the body, and in both the acute and post-acute phases [70]. Pulmonary rehabilitation plays an important role in the long COVID patient. A meta-analysis by Meléndez-Oliva et al. examined the impact of pulmonary rehabilitation in patients with subacute and prolonged COVID-19 infections [71]. They assessed the improvements in dyspnea, physical function, quality of life, psychological status (anxiety and depression), and fatigue. They demonstrated that pulmonary rehabilitation has the potential to improve various health outcomes in patients, including those recovering from COVID-19. Pulmonary rehabilitation showed positive effects on dyspnea, physical function, quality of life, and depressive symptoms compared to standard interventions [72]. Elyazed et al. investigated the effect of home pulmonary rehabilitation on exercise capacity in patients with post-COVID-19 syndrome [73]. The control group received only the usual medical care, while the rehabilitation group received a selected home pulmonary rehabilitation exercise program in addition to the same usual medical care. They showed that home pulmonary rehabilitation is effective and has a positive effect on exercise capacity, fatigue, dyspnea, and quality of life. They point out that home pulmonary rehabilitation could be considered as an adjunctive, applicable, and low-cost therapy for patients with post-COVID-19 syndrome [71]. Recently, Li et al. performed a meta-analysis of 37 studies to evaluate pulmonary rehabilitation in patients with long-standing COVID-19 [74]. They found that a pulmonary rehabilitation program lasting 4–8 weeks and a combination of breathing exercises with multicomponent training are most effective for the management of long-standing COVID-19 syndromes. The results of this meta-analysis found that pulmonary rehabilitation significantly improved exercise capacity, respiratory function, health-related quality of life (HRQoL), fatigue, and anxiety in patients with long-standing COVID-19. Early initiation of rehabilitation and a 4–8 week duration are important in the management of prolonged COVID-19 syndromes [73]. The reporting rate and severity of COVID-19 have declined over time. Timely interventions are needed to minimize the impact of long COVID, especially for older adults, women, and those with SARS-CoV-2 reinfection. Booster vaccinations against COVID-19 could play a potential role in minimizing the impact of long COVID in conjunction with pulmonary rehabilitation.

**Figure 1:**
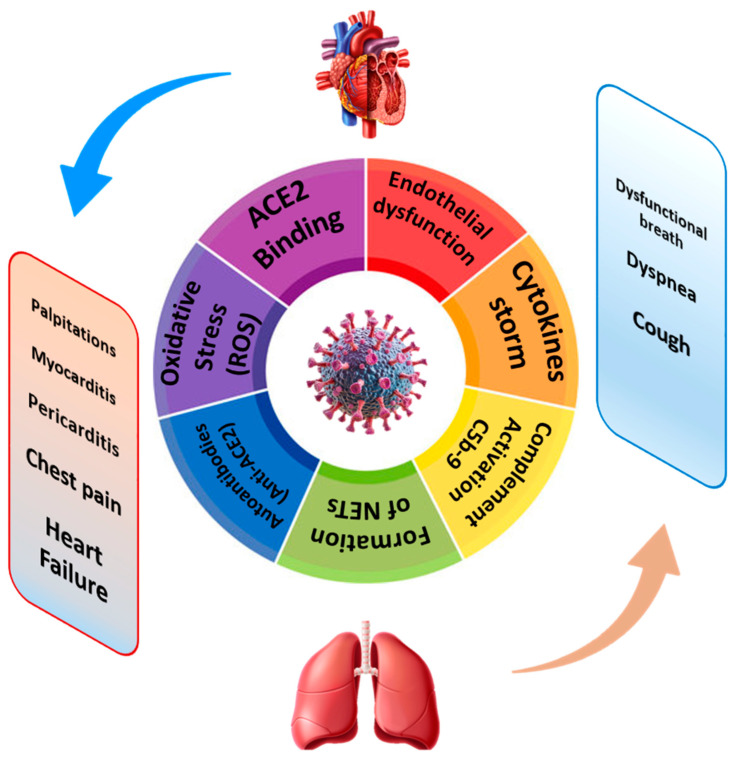
Schematic view of cardio-pulmonary long COVID: from bench to bedside.

A summary view of the cardio-pulmonary involvement in long COVID is provided in Figure 1.

## 6. Conclusions

Cardio-pulmonary complications in long COVID result from a multifactorial interplay of immune dysregulation, endothelial injury, and persistent tissue inflammation. Myocardial damage, including subclinical or overt myocarditis, is frequently associated with pulmonary impairment—ranging from reduced diffusion capacity to fibrotic remodeling—suggesting a shared inflammatory and microvascular pathology [2,53]. Persistent low-grade inflammation, immune-mediated injury, and microthrombosis represent the core mechanisms underlying these changes, often exacerbated by viral persistence and endothelial dysfunction [18,55]. Clinically, this cardio-pulmonary axis contributes to a heterogeneous spectrum of symptoms, including dyspnea on exertion, chest pain, tachycardia, desaturation, and a reduced exercise capacity. The overlap between the cardiac and pulmonary symptoms necessitates a multidisciplinary care model, involving cardiologists, pulmonologists, hematologists, immunologists, and rehabilitation experts, to ensure a comprehensive evaluation and targeted interventions [54].

Moreover, the prothrombotic state observed in many long COVID patients, including evidence of persistent platelet activation, elevated D-dimers, and microvascular thromboses, underscores the need for individualized risk assessments and possibly extended thromboprophylaxis in select patients [74]. Preventive interventions such as vitamin D supplementation—which has shown immunomodulatory and endothelial protective effects—and the judicious use of anticoagulants are being explored as strategies to reduce post-COVID sequelae, although robust clinical evidence is still needed [75].

Looking forward, longitudinal studies with histopathological correlation are essential to clarify the natural history of myocardial and pulmonary recovery or progression to fibrosis and dysfunction. Advanced imaging, serial biomarker assessment, and molecular profiling will be crucial in identifying the therapeutic targets. Particular attention should be paid to immune–fibrotic pathways, viral reservoirs, and endothelial–immune cross-talk, which may open avenues for disease-modifying therapies beyond symptom management.

In summary, long COVID represents a complex cardio-pulmonary syndrome that extends beyond acute infection. It requires a multidimensional approach combining early diagnosis, targeted prevention, and personalized rehabilitation. As the global burden of long COVID grows, translational research efforts must accelerate to improve the long-term outcomes for affected patients.

### Limitations and Strengths

This study has several important limitations that should be acknowledged. Firstly, the heterogeneous nature of the available data on long COVID syndrome, particularly regarding its cardio-pulmonary manifestations, limits the generalizability of the findings. Additionally, most of the referenced studies are observational cohorts with variable follow-up durations and often lack adequate control for the confounding factors such as pre-existing comorbidities and the severity of the acute infection. The absence of standardized biomarker data and advanced imaging across large populations reduces the ability to precisely identify the pathophysiological mechanisms and long-term outcome predictors. Furthermore, evidence on the efficacy of specific therapeutic interventions remains limited, highlighting the need for randomized controlled trials.

**Figure 2:**
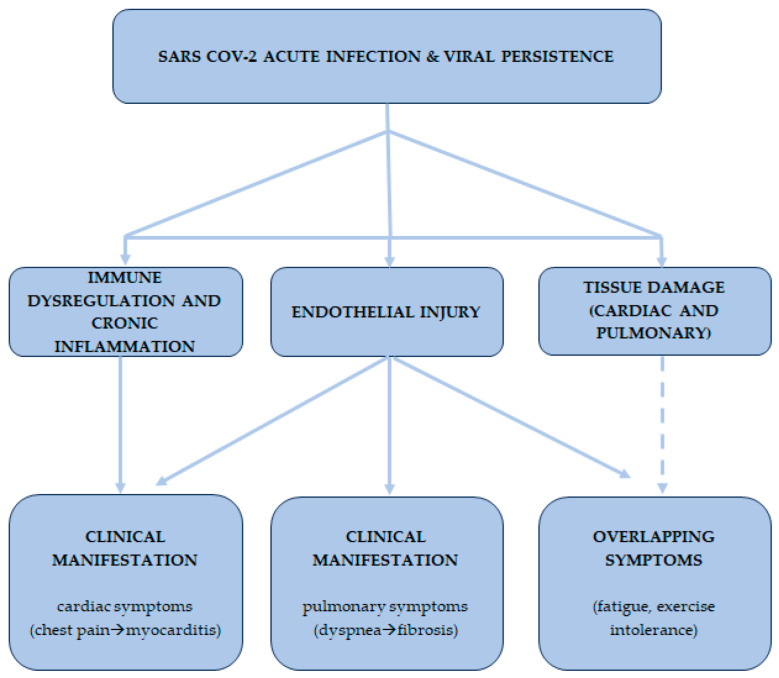
This conceptual diagram illustrates the complex, multifactorial pathophysiology of the cardio-pulmonary sequelae in long COVID. The model begins with acute SARS-CoV-2 infection, which triggers a cascade of core mechanisms, including immune dysregulation, endothelial injury, and tissue damage. Solid arrows represent direct, well-documented relationships between these mechanisms and their specific clinical manifestations. Dashed arrows, in contrast, denote less direct or convergent pathways, such as the contributions of widespread endothelial and tissue damage to overlapping symptoms like fatigue and exercise intolerance. This framework highlights the interconnectedness of cardiac and pulmonary pathology, emphasizing the need for an integrated, multidisciplinary diagnostic and therapeutic approach.

Nonetheless, this work also presents notable strengths. It provides a comprehensive synthesis of the immuno-fibrotic, microvascular, and inflammatory mechanisms underpinning both the cardiac and pulmonary involvement in long COVID as reported in Figure 2, emphasizing the need for a multidisciplinary clinical approach. The focus on advanced diagnostic modalities, such as cardiac magnetic resonance imaging and high-resolution computed tomography, allows for a more precise characterization of the pathological continuum from acute infection to chronic sequelae. Moreover, the article underscores the critical need for longitudinal studies and molecular biomarker profiling to develop targeted therapies, thereby outlining a clear roadmap for future research. This integrated perspective represents a significant contribution to the understanding and management of post-COVID cardio-pulmonary complications.

## Figures and Tables

**Table 1:** Summary Table—long COVID: molecular, pathophysiological, and clinical aspects.

**Molecular aspects**	
Endothelial dysfunction	Reduced nitric oxide (NO), oxidative stress via NOX2, upregulation of adhesion molecules (VCAM-1, ICAM-1), elevated von Willebrand factor (vWF), angiopoietin-2 (Ang-2), soluble thrombomodulin
Chronic inflammation	Persistent elevation of IL-6, IL-1β, TNF-α, CRP; activation of inflammatory pathways (NF-κB, JAK/STAT, MAPK)
Immune system overactivation	Ongoing activation of CD14^+^ monocytes, persistent dysregulated CD8^+^ T-cells, dysfunctional NK cells
Autoimmunity	Presence of autoantibodies against GPCRs, ACE2, endothelial antigens, adrenergic and muscarinic receptors
Molecular mimicry	Cross-reactivity between viral antigens and host cardiac proteins leading to immune attack
Epigenetic & metabolic dysfunction	Mitochondrial impairment, altered microRNA expression, sustained activation of interferon-stimulated genes
Fibrotic remodeling	Activation of TGF-β pathway promoting fibrosis in lungs and heart
Prothrombotic state	Elevated PAI-1, Factor VIII, tissue factor; platelet hyperactivation and persistent D-dimer levels; resistant microthrombi
**Pathophysiological aspects**	
Cardiac damage	Myocarditis, subclinical inflammation, myocardial fibrosis, reduced ventricular compliance, arrhythmias
Pulmonary involvement	Interstitial lung disease, alveolar damage, persistent inflammation, fibrosis, microangiopathy
Vascular injury	Endothelial inflammation and dysfunction, microvascular thrombosis in multiple organs
Dysautonomia	Autonomic nervous system imbalance: inappropriate sinus tachycardia, low blood pressure, impaired vascular tone
Viral persistence	Detection of SARS-CoV-2 RNA or proteins in myocardial or lung tissue months after infection
**Clinical aspects**	
Cardio-pulmonary symptoms	Fatigue, dyspnea on exertion, chest pain, desaturation, palpitations, exercise intolerance
Systemic symptoms	Cognitive dysfunction (“brain fog”), anxiety, depression, sleep disorders, arthralgia
Vascular complications	Risk of late thromboembolic events (e.g., pulmonary embolism, stroke), subclinical ischemia

## Data Availability

The data from this manuscript are derived from publicly available published clinical trial and study results.
